# Biosynthesis of the proteasome inhibitor syringolin A: the ureido group joining two amino acids originates from bicarbonate

**DOI:** 10.1186/1471-2091-10-26

**Published:** 2009-10-28

**Authors:** Christina Ramel, Micha Tobler, Martin Meyer, Laurent Bigler, Marc-Olivier Ebert, Barbara Schellenberg, Robert Dudler

**Affiliations:** 1 Institute of Plant Biology and Zurich-Basel Plant Science Center, University of Zurich, Zurich, Switzerland; 2 Institute of Organic Chemistry, University of Zurich, Zurich, Switzerland; 3 Laboratory of Organic Chemistry, ETH Zurich, Zurich, Switzerland

## Abstract

**Background:**

Syringolin A, an important virulence factor in the interaction of the phytopathogenic bacterium *Pseudomonas syringae *pv. *syringae *B728a with its host plant *Phaseolus vulgaris *(bean), was recently shown to irreversibly inhibit eukaryotic proteasomes by a novel mechanism. Syringolin A is synthesized by a mixed non-ribosomal peptide synthetase/polyketide synthetase and consists of a tripeptide part including a twelve-membered ring with an N-terminal valine that is joined to a second valine via a very unusual ureido group. Analysis of sequence and architecture of the syringolin A synthetase gene cluster with the five open reading frames *sylA-sylE *allowed to formulate a biosynthesis model that explained all structural features of the tripeptide part of syringolin A but left the biosynthesis of the unusual ureido group unaccounted for.

**Results:**

We have cloned a 22 kb genomic fragment containing the *sylA-sylE *gene cluster but no other complete gene into the broad host range cosmid pLAFR3. Transfer of the recombinant cosmid into *Pseudomonas putida *and *P. syringae *pv. *syringae *SM was sufficient to direct the biosynthesis of *bona fide *syringolin A in these heterologous organisms whose genomes do not contain homologous genes. NMR analysis of syringolin A isolated from cultures grown in the presence of NaH^13^CO_3 _revealed preferential ^13^C-labeling at the ureido carbonyl position.

**Conclusion:**

The results show that no additional syringolin A-specific genes were needed for the biosynthesis of the enigmatic ureido group joining two amino acids. They reveal the source of the ureido carbonyl group to be bicarbonate/carbon dioxide, which we hypothesize is incorporated by carbamylation of valine mediated by the *sylC *gene product(s). A similar mechanism may also play a role in the biosynthesis of other ureido-group-containing NRPS products known largely from cyanobacteria.

## Background

Syringolins are a family of closely related cyclic peptide derivatives that are secreted by many strains of the phytopathogenic bacterium *Pseudomonas syringae *pv. *syringae *(*Pss) in planta *and under certain culture conditions [1,2]. Syringolin A, the major variant, was shown not only to induce acquired resistance in rice and wheat after spray application, but also to trigger hypersensitive cell death at infection sites of wheat and Arabidopsis plants infected by compatible powdery mildew fungi [3,4]. Recently, syringolin A was shown to be an important virulence factor in the interaction of *Pss *B728a with its host plant *Phaseolus vulgaris *(bean), and its cellular target has been identified. Syringolin A irreversibly inhibits the eukaryotic proteasome by a novel mechanism, representing a new structural class of proteasome inhibitors [5,6].

**Figure 1:**
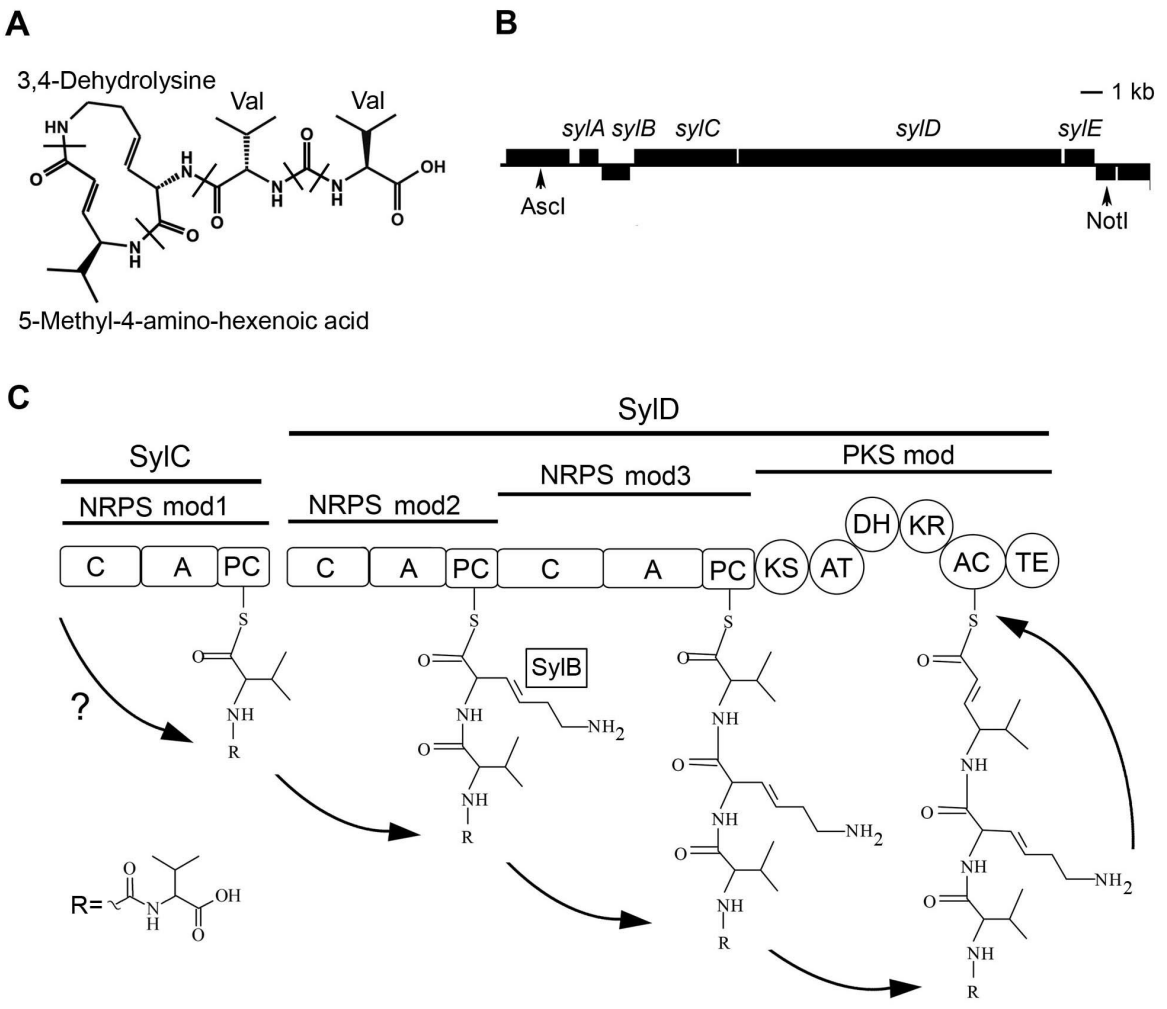
**Structure and biosynthesis model of syringolin A**. A. Structure of syringolin A. Amino acid constituents are delimited by bars. Val, valine. B. Genomic region of *Pss *B301D-R containing the *sylA-sylE *genes. Boxes above and below the line denote ORFs on the top and the bottom strand, respectively. Arrows indicate restriction sites used for cloning of the gene cluster into the cosmid pPL3syl. The *sylA, sylB*, and *sylE *genes encode a LuxR-type transcription activator, a rhizobitoxin desaturase-like protein thought to desaturate the lysine residue, and an efflux transporter, respectively [10]. The *sylC *gene encodes an NRPS module, while *sylD *codes for two NRPS modules and one PKS module [10] C. Biosynthesis model of the tripeptide part of syringolin A. The open boxes represent domains in modules of the syringolin A synthetase labeled with C, condensation domain; A, adenylation domain; PC, peptide carrier protein; KS, ketoacyl synthase; AT, acyl transferase; DH, dehydratase; KR, ketoreductase; AC, acyl carrier protein; TE, thioesterase. The A domains of the NRPS modules are thought to activate valine (NRPS mod1), lysine (NRPS mod2), and valine (NRPS mod3) [10]. The question mark indicates the unexplained synthesis and attachment of this group. The figures are adapted from [10].

Structure elucidation revealed that syringolin A is a tripeptide derivative consisting of an N-terminal valine followed by the two non-proteinogenic amino acids 3,4-dehydrolysine and 5-methyl-4-amino-2-hexenoic acid, the latter two forming a twelve-membered macrolactam ring. The N-terminal valine is in turn linked to a second valine via an unusual ureido group (Figure 1A; [1]). The minor variants syringolin B to syringolin F differ from syringolin A by the substitution of one or both valines with isoleucine residues, by the substitution of 3,4-dehydrolysine with lysine, and by combinations thereof [2]. The structure of syringolin A suggested that it was synthesized by a non-ribosomal peptide synthetase (NRPS), large modular enzymes that activate and condense amino acids according to the thiotemplate mechanism (for reviews see e.g. [7-9]). We previously cloned and delimited by mutational analysis a genomic region from *Pss *B301D-R containing five open reading frames (*sylA-sylE*) necessary for syringolin biosynthesis (Figure 1B; [10]). Whereas *sylA *and *sylE *encode a putative transcription activator and an exporter, respectively, *sylC *encodes a typical NRPS module predicted to activate valine, whereas *sylD *codes for two additional NRPS modules (of which the first is predicted to activate lysine and the second is predicted to activate valine [10]) and a type I polyketide synthetase (PKS) module. Type I PKS are also modular enzymes that, similar to fatty acid synthesis, extend a starter molecule by condensation/decarboxylation of malonate extender units (for reviews see e.g. [11,12]). The analysis of the structure and architecture of the *syl *gene cluster led to the postulation of a model that completely accounts for the biosynthesis of the tripeptide part of syringolin A, including its ring structure with the 5-methyl-4-amino-2-hexenoic acid and the 3,4-dehydrolysine (Figure 1C, [10]). However, although the addition of the ureido group and its attached second valine could not be explained by the model, the *syl *gene cluster did not contain additional open reading frames, which, if present, could potentially have been involved in the biosynthesis of this unexplained part.

Here we show that the genes *sylA-sylE *are sufficient to direct the biosynthesis of *bona fide *syringolin A when heterologously expressed in *Pseudomonas putida *and *Pss *SM, two organisms which do not produce syringolin A and have no *syl *gene cluster homolog in their genomes. Thus, biosynthesis of the ureido group with its attached terminal valine is achieved without additional syringolin A-specific genes (*i. e*. genes with no other function than in syringolin A biosynthesis). We hypothesized that biosynthesis of the ureido group would most likely be accomplished by the product of the *sylC *gene, which would, in addition to the extracyclic peptidyl valine, also activate the terminal valine and join the two residues by incorporation of a carbonyl group derived from hydrogen carbonate/carbon dioxide, thus forming the ureido moiety. We demonstrate by NMR spectroscopic analysis of syringolin A isolated from *Pss *cultures grown in the presence of NaH^13^CO_3 _that the ^13^C isotope is preferentially found at the position of the ureido carbonyl atom. These results support our hypothesis, which may be of relevance for the hitherto unknown biosynthesis of other ureido-group-containing NRPS products largely known to be produced by cyanobacteria [13-20].

## Results

### Biosynthesis of syringolin A in heterologous organisms

In order to test whether the *sylA-sylE *gene cluster was sufficient to direct syringolin A biosynthesis, we constructed a cosmid containing the *sylA-sylE *genes but no other complete gene by taking advantage of *Asc*I and *Not*I restriction sites flanking the *syl *gene cluster (Figure 1B). Southern blot analysis of *Asc*I/*Not*I-digested genomic DNA of *Pss *B301D-R probed with a *sylA *gene fragment labeled the expected 22 kb fragment and thus confirmed the uniqueness of the restriction sites in the relevant genome region (data not shown). Thus, *Pss *B301D-R genomic DNA digested with *Asc*I and *Not*I was separated by agarose gel electrophoresis. Fragments in the 20-23 kb size range were eluted and cloned into the wide host range cosmid pLAFR3 [21]. After packaging into lambda phages and transfection into *E. coli *XL-1Blue, the library was screened with a radiolabeled *sylA *gene probe. Positive clones were isolated and confirmed to contain the complete *syl *gene cluster by PCR amplification and sequencing of the expected insert ends. One of the confirmed clones was designated pPL3syl and chosen for further work.

**Figure 2:**
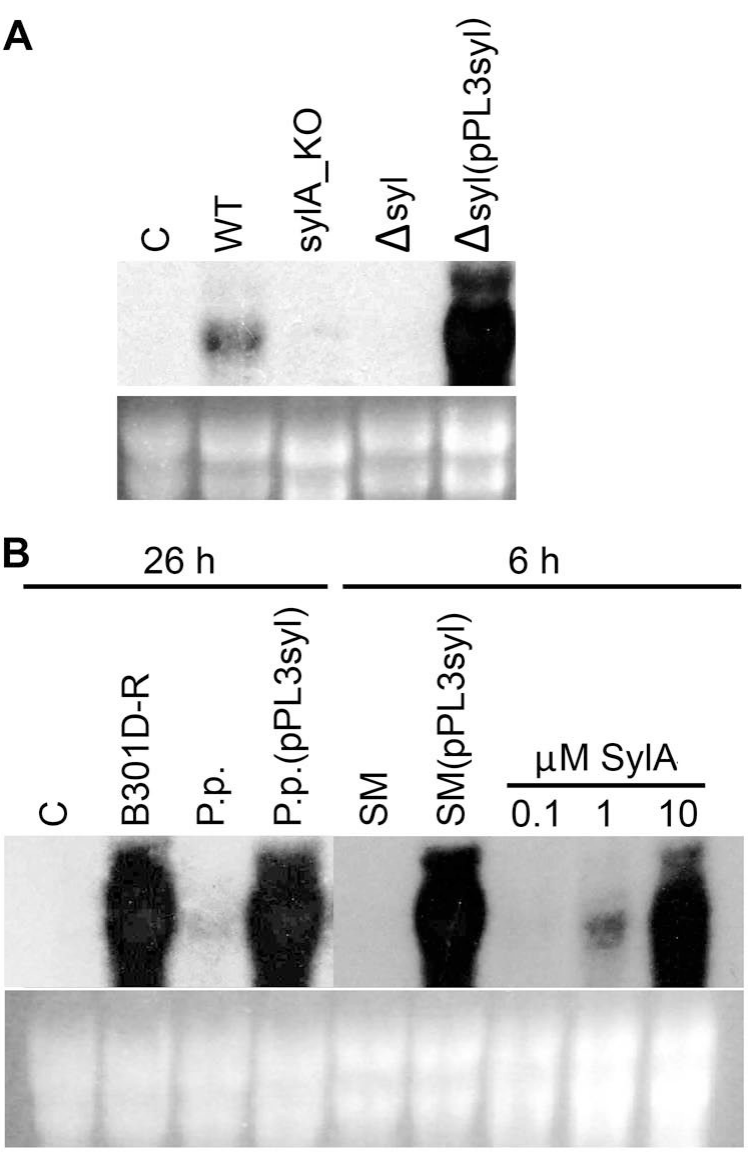
**Gel blot analysis of *Pir7b *transcript accumulation**. A. RNA was extracted from rice leaves infiltrated with water (C), *Pss *B301D-R (WT), a *sylA *plasmid insertion mutant (sylA_KO), a *syl *cluster deletion mutant (Δsyl), and Δsyl (pPL3syl), the deletion mutant complemented with the wild-type *syl *gene cluster. Top panel, autoradiogram (exposed for 5 h); bottom panel, ethidium bromide (EtBr)-stained agarose gel. B. Lanes were loaded with RNA extracted from rice leaves infiltrated as indicated. C, water control; B301D-R, *Pss *wild-type strain; P.p, *P. putida *P3; P.p. (pPL3syl), *P. putida *P3 transformed with the *syl *gene cluster; SM, *Pss *SM; SM (pPL3syl); *Pss *SM transformed with pPL3syl, and syringolin A solutions of the indicated concentrations. Top panel, autoradiogram (exposure times indicated on top), bottom panel, EtBr-stained gel.

To test the functionality of pPL3syl, the markerless *Pss *B301D-R mutant Δsyl was constructed in which the complete *syl *gene cluster was deleted. The pPL3syl cosmid was then mobilized into the Δsyl deletion mutant by triparental mating. We previously showed that infiltration of syringolin A-producing *Pss *strains or isolated syringolin A into rice leaves leads to the accumulation of transcripts corresponding to the defense-related *Pir7b *gene (encoding an esterase; [22]), whereas strains or mutants unable to synthesize syringolin A do not activate this gene [1,23]. Syringolin A was originally identified and isolated based on its action on the *Pir7b *gene in rice [1]. We thus infiltrated the B301D-R wild-type strain, the syringolin-negative mutants Δsyl and sylA_KO (contains a plasmid insertion interrupting the *sylA *transcription activator gene [10]), as well as Δsyl (pPL3syl), the deletion mutant complemented with pPL3syl, into rice leaves. RNA was extracted and subjected to gel blot analysis with regard to *Pir7b *transcript accumulation. As expected and in contrast to the wild type, the syringolin A-negative mutants did not induce *Pir7b *transcript accumulation, whereas the deletion mutant complemented with the pPL3syl cosmid led to a much stronger induction of the *Pir7b *gene (Figure 2A). This strongly suggested that pPL3syl contained a functional *syl *gene cluster able to direct syringolin A synthesis in the Δsyl deletion mutant. This does not exclude the possibility that genes not present in the *syl *gene cluster are necessary for syringolin A production because such genes would also be present in the Δsyl mutant background.

Next we wanted to mobilize pPL3syl into *Pseudomonas *strains not carrying *syl *gene homologs and lacking syringolin A production as evidenced by PCR, DNA gel blot analysis of genomic DNA, high performance liquid chromatography (HPLC) analysis of culture supernatants with regard to syringolin A content, infiltration into rice leaves followed by monitoring of *Pir7b *transcript accumulation, and whole genome sequence comparisons where possible (data not shown). After repeated unsuccessful attempts to transfer pPL3syl into the *P. syringae *pv. *tomato *DC3000 strain (all tetracycline-resistant putative transformants analyzed contained deletion variants of pPL3syl), the cosmid was successfully transferred into the non-pathogenic bacterium *P. putida *P3 [24] and *Pss *SM, a strain originally isolated from wheat [23,25]. Gel blot analysis of RNA extracted from rice leaves infiltrated with parental and transformed strains showed that, as expected, *P. putida *P3 and *Pss *SM did not induce *Pir7b *transcript accumulation. In contrast, both strains lead to *Pir7b *gene induction when carrying the pPL3syl cosmid (Figure 2B), suggesting that pPL3syl conferred the ability for syringolin A biosynthesis to these strains.

**Figure 3:**
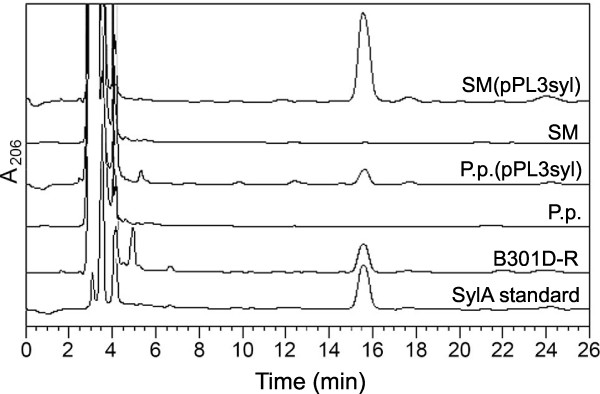
**HPLC analysis of syringolin A content in conditioned SRM_AF _media**. Conditioned media were sterile-filtrated and 20-μl-aliquots were loaded on the column. Absorption was monitored at 206 nm. Labels of HPLC traces are the same as in the legend to Figure 2B.

To confirm this, the transformed strains were grown in shaken cultures in SRM_AF _medium and conditioned media were analyzed by HPLC. As shown in Figure 3, both strains produced a compound eluting at 15.5 min, the elution time of the syringolin A standard. Peaks were collected from multiple HPLC runs and subjected to mass spectrometry. HPLC-high resolution-electrospray ionization-mass spectrometry (HPLC-HR-ESI-MS) of the peaks from *Pss *SM and *P. putida *P3 carrying pPL3syl, and the *Pss *B301D-R wild type showed quasi-molecular ions [M+H]^+ ^at *m/z *494.29808 (1.5 ppm difference from calculated exact mass), 494.29653 (1.1 ppm), and 494.29799 (1.4 ppm), respectively, matching the empirical formula C_24_H_40_N_5_O_6_^+ ^(protonated adduct of syringolin A; calculated exact mass 494.29731). We conclude from these experiments that the *syl *genes contained in pPL3syl are sufficient to direct syringolin A biosynthesis in these heterologous strains and no further syringolin A-specific genes, *i.e*. genes that exclusively function in syringolin A biosynthesis, are necessary.

### The ureido carbonyl group of syringolin A is incorporated from bicarbonate/carbon dioxide

The above results raised the question of how the ureido-valine is synthesized and incorporated into syringolin A. We hypothesized that this would most likely be accomplished by the product of the *sylC *gene, which would, in addition to the N-terminal valine of the tripeptide part of syringolin A, also activate the second valine and join the two residues via their amino groups formally by amidation of carbonic acid, thus forming the ureido moiety. If true, feeding syringolin A-producing cultures with ^13^C-labeled hydrogen carbonate should result in syringolin A that is preferentially labeled with ^13^C at the ureido carbonyl position. Thus, *Pss *B301D-R transformed with pOEAC, a plasmid carrying the *sylA *transcriptional activator gene under the control of the *lacZ *promoter, was grown in SRM_AF _medium. After 48 h, ^13^C-labeled sodium hydrogen carbonate was added to a final concentration of 70 mM and the culture was further grown for 20 h. Syringolin A was isolated from conditioned medium as described [4] and subjected to ^13^C NMR analysis.

**Figure 4:**
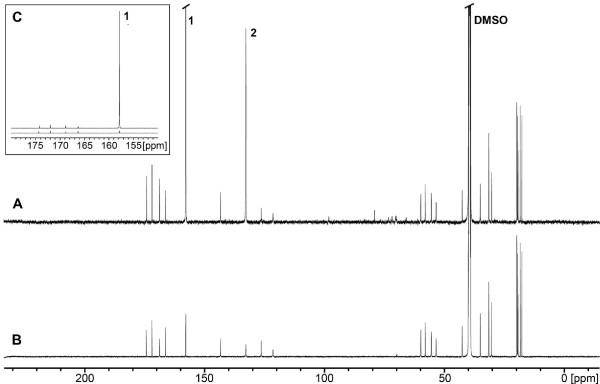
**^13^C-NMR spectra of *in vivo *NaH^13^CO_3_-labeled and unlabeled syringolin A**. The spectra of NaH^13^CO_3_-labeled (A) and unlabeled (B) syringolin A have been scaled to give equal signal intensities for the methyl groups of the valine residues (17.4, 17.5, 19.0, and 19.1 ppm shifts). The signals at 157.8 ppm (marked 1; clipped off) and 132.8 ppm (marked 2) correspond to the ureido carbonyl group and the lysine C4 position, respectively. DMSO, DMSO solvent signal. C. Scaled-down version of part of the spectra given in (A) and (B) to show the difference in signal intensity of the ureido carbonyl group in NaH^13^CO_3_-labeled and unlabeled syringolin A, respectively.

The spectrum of labeled syringolin A was normalized in order to get the same signal intensities for the valine methyl groups as in the unlabeled sample. Comparison of the normalized NMR spectra revealed that the signal from the ureido carbon atom in ^13^C-labeled syringolin A was 45-fold stronger than the corresponding signal from unlabeled syringolin A (Figure 4). Inspection of the resolved ^13^C satellite of the valine methyl group at lowest field in the ^1^H spectrum of labeled syringolin A (data not shown) suggests a ^13^C content close to natural abundance. Therefore, the 45-fold signal enhancement in labeled syringolin A directly corresponds to the absolute ^13^C enrichment at this site. The normalized signal strengths of all other C atoms were equal in labeled and unlabeled syringolin A, with the exception of the C4 position of 3,4-dehydrolysine, whose signal was enhanced approximately 16-fold in ^13^C-labeled syringolin A (Figure 4A, B). Inspection of biosynthetic pathways using the KEGG database [26] revealed that this can be attributed to a carboxylation reaction in the biosynthesis of lysine. The C4 atom of lysine represents the C4 atom of L-aspartate-4-semialdehyde, a derivative of aspartate, which is condensed to pyruvate to yield the intermediary compound L-2,3-dihydrodipicolinate in bacterial lysine biosynthesis. The C4 atom of aspartate in turn originates from the carboxylation of pyruvate to oxaloacetate, an intermediary compound in the tricarboxylic acid cycle, which is transaminated to aspartate. Thus, enhanced ^13^C-labeling of lysine with H^13^CO_3_^- ^at the C4 position is to be expected. We note that malonate will also be labeled by H^13^CO_3_^- ^as it is derived from acetate by carboxylation. However, the label will be removed by the condensation/decarboxylation of malonate to the peptide chain during syringolin A biosynthesis. We conclude from this analysis that our hypothesis is correct, *i.e*. that the ureido carbonyl moiety in syringolin A originates from the incorporation of hydrogen carbonate/carbon dioxide.

## Discussion

We have demonstrated that the *syl *gene cluster is sufficient to direct syringolin A synthesis in heterologous organisms. Although the biosynthesis model presented earlier [10] plausibly explained every structural feature of the syringolin A tripeptide part through the enzymatic actions of the *sylB, sylC*, and *sylD *gene products, the generation and condensation of the ureido valine remained enigmatic. As the other genes present in the *syl *cluster encode a transcriptional activator (*sylA *gene) and an exporter (*sylE *gene), a plausible hypothesis was that the *sylC*-encoded NRPS module not only activated the N-terminal peptidyl valine, but also the ureido valine, and that the ureido carbonyl moiety is incorporated from hydrogen carbonate/carbon dioxide. As shown above, *in vivo *labeling of syringolin A with ^13^C-hydrogen carbonate supports this hypothesis. Currently, we can only speculate how this is achieved. One possibility is that the quaternary syringolin A synthetase complex may contain two (not necessarily identical) molecules derived from the *sylC *gene per SylD polypeptide. Both *sylC *gene products would activate valine, or, to a certain degree, isoleucine in minor syringolin variants [2]. The first valine would then be carbamylated by HCO_3_^-^/CO_2_, perhaps without the action of another enzyme, as has been reported for the carbamylation of a catalytic lysine residue in β-lactamases of class D [27,28]. The ureido moiety would then be formed by amide bond formation between the carbamylated valine and the second valine. In this scenario, it remains unclear how the first valine, which, like the second one, is envisioned to be bound to the peptide carrier protein domain by a thioester bond, is released upon ureido bond formation. It is also conceivable that ureido bond formation is achieved by a single SylC protein, which contains a condensation domain usually absent from starter modules that may be involved. To clarify these issues, more structural information about the large syringolin A synthetase and the SylC module must be obtained. The reconstitution of the enzymatic activities of the module(s) derived from the *sylC *gene *in vitro *will be challenging.

In addition to the syringolin family of compounds, a number of other natural cyclic peptides mostly isolated from cyanobacteria have been described in the literature that contain extracyclic ureido groups linking two different amino acids. These include anabaenopeptins from *Anabaena, Oscillatoria*, and *Planktothrix *species [13-16], ferintoic acids from *Mycrocystis aeruginosa *[17], pompanopeptins from *Lyngbya confervoides *[18], as well as mozamides and brunsvicamides, compounds of presumably cyanobacterial origin isolated from sponges [19,20]. Bicarbonate/CO_2 _may also be the source of the ureido carbonyl group joining two extracyclic amino acids in the biosynthesis of these compounds, which, to our knowledge, has not been elucidated so far.

## Conclusion

Our results show that the *syl *biosynthesis gene cluster was sufficient to direct the biosynthesis of *bona fide *syringolin A, including the enigmatic ureido group joining two amino acids. They reveal the source of the ureido carbonyl group to be bicarbonate/carbon dioxide, which we hypothesize is incorporated by carbamylation of valine mediated by the *sylC *gene product(s). A similar mechanism may also play a role in the biosynthesis of other ureido-group-containing NRPS products known largely from cyanobacteria.

## Methods

### Construction and expression of pPL3syl

Unless stated otherwise, standard protocols were used [29]. Genomic DNA from *Pss *B301D-R was isolated and 11 μg were digested with the restriction enzymes *Asc*I and *Not*I. *Asc*I and *Not*I sites are both unique in the *syl *gene region (GenBank: AJ548826) located at position 2052 and 24124, respectively, within the ORFs flanking the *sylA*-*sylE *ORFs (3507-23596). A DNA gel blot was prepared with 1 μg of the digested DNA and probed with a ^32^P-labeled *sylA *gene fragment PCR-amplified from genomic DNA with primers P1 (5'-ccatcgatggagtagagtgatggc) and P2 (5'-ggaattcttacaaaattcccatcttg). The rest of the digested DNA was separated on a 0.4% agarose gel and the DNA in the 20-23 kb size range was cut out, electrophoretically eluted into a dialysis bag (10 kDa cutoff), extracted first with 1 volume of phenol and then with 1 volume of phenol-chloroform-isoamylalcohol (25:24:1), precipitated with ethanol and finally taken up in TE (10 mM Tris-HCl, pH 8; 1 mM EDTA). Fragments were ligated into the *Hin*dIII/*Bam*HI-cut broad host range cosmid vector pLAFR3 [21] using adaptors prepared by annealing the oligonucleotide 5'-cgcgccaagcttcca with 5'-agcttggaaagcttgg (*Asc*I/*Hin*dIII adaptor) and 5'-ggccgctagtcaggag with 5'-gatcctcctgactagc (*Not*I/*Bam*HI adaptor), respectively. Ligation products were packaged into lambda phage particles using the Gigapack III Gold Packaging Kit (Stratagene, La Jolla, California) and the library was plated out on *E. coli *XL1-Blue (Stratagene) and screened according to the instructions of the manufacturer using the ^32^P-labeled *sylA *gene fragment described above as a probe. Positive clones were isolated and confirmed to contain the complete *syl *gene cluster by PCR amplification and sequencing of the insert end fragments using primers 5'-ccggcctacacgcattc (*sylA *end) and 5'-agcaacctggatgtacgg (*sylE *end) with the respective adaptor oligonucleotides (see above).

pPL3syl was transferred from XL1-Blue to *Pseudomonas *strains by triparental mating using the *E. coli *helper strain HB101 (pRK600) [30,31].

### Construction of the *syl *gene cluster deletion mutant Δsyl

Two fragments of 783 bp and 655 bp length flanking the *syl *gene cluster on the 5' and 3' side, respectively, were amplified by PCR from *Pss *B301D-R genomic DNA using the primer pairs P3 (5'-cgggatcc**a**acctgaaatgggagagtc; base given in bold at position 2297 in GenBank:AJ548826) and P4 (5'-agcgcgagga**c**tcaatgtgaaaacaacg; bold base at position 3072), and P5 (5'-tcacattgag**t**cctcgcgctggtaacc; bold base at position 23600) and P6 (5'-**t**tctgcagtcaagcctgacgaaaagc; bold base at position 24247), respectively. The two bands were isolated and joined by overlap extension PCR using primers P3 and P6 to yield a fragment flanked by *Bam*HI and *Pst*I restriction sites in which the *syl *gene cluster from position 3073-23599 (GenBank:AJ548826) was missing. The deletion is nearly identical with the one in the completely sequenced *P. syringae *pv. *tomato *DC3000 (GenBank:NC_004578.1), which does not contain a *syl *gene cluster. The fragment was cut with *Bam*HI and *Pst*I and cloned into the respective restriction sites in the cloning box of the suicide vector pME3087 (Tc^R^, ColE1 replicon [32]). The recombinant plasmid was transformed into *E. coli *S17-1 (*thi pro hsdR recA*; chromosomal RP4 (Tra^+ ^Tc^S ^Km^S ^Ap^S^; transfer gene-positive, tetracycline-sensitive, kanamycin-sensitive, ampicillin-sensitive) [33]) and mobilized into *Pss *B301D-R. Tetracycline-resistant colonies were grown in LB medium over night at 28°C on a rotary shaker (220 rpm). For selection of tetracycline-sensitive colonies, the overnight cultures were diluted 100-fold with LB. After 2 h of growth, tetracycline was added (20 μg/ml final concentration) and the cultures were grown for 1 h, after which the bactericide carbenicillin (2 mg/ml final concentration) was added for 3 h. The bacteria were then collected by centrifugation, and after washing them twice in LB, the selection procedure was repeated another 3 times. The cultures were then replica-plated on LB plates with and without tetracycline (10 μg/ml) and tetracycline-sensitive colonies were isolated (about 2-3%). The desired deletion mutants were distinguished from wild-type revertants and verified by sequencing of a 1.7 kb DNA fragment amplified from genomic DNA by PCR using primers 5'-attactcgaccagttccg and 5'-ttacgcaatggtatgatgc which are located outside the fragment cloned into the suicide vector pME3087 at position 2113 and 24385 (GenBank:NC_007005.1), respectively.

### Construction of pOEAC

The *sylA *ORF was amplified from genomic DNA using the primers P7 (5'-cc*atcgat*ggagtagagtg**atg**gc; *Cla*I site in italics, translation initiation codon indicated in bold) and P8 (5'-g*gaattc***tta**caaaattcccatcttg; reverse primer; *Eco*RI site in italics, reverse stop codon in bold), digested with *Cla*I and *Eco*RI, and cloned into the respective polylinker sites of the pME6001 (Gm^R^) expression vector [34], thereby placing it under the control of the *lacZ *promoter. The resulting plasmid was named pOEA. As it turned out that pOEA did not confer gentamycin resistance in SRM_AF _medium, pOEAC was used, a derivative of pME6014 (Tet^R^) [35], which, in addition to the *lacZ::sylA *chimeric gene, contained a *sylC::lacZ *reporter fusion gene in opposite orientation (the reporter gene is of no relevance in the present context). To construct pOEAC, the *lacZ::sylA *fusion gene was amplified from pOEA with primers P8 (see above) and P9 (5'-accgtccaac**a**ttaatgcagctgg; upstream of *lac *promoter; bold base complementary to position 987 of pBluescript vector (GenBank:X52329)) and joined with a *sylC *promoter fragment (position 5409-5649 of GenBank:AJ548826) that was amplified with primers P10 (5'-ctgcattaat**g**ttggacggtctgc; bold base at position 5409) and P11 (5'-aa*ctgcag***t**catgacggcctcggat; *Pst*I site in italics, bold base at position 5649) by overlap extension PCR using primers P8 and P11. The resulting fragment was digested with *Eco*RI and *Pst*I and cloned between the respective sites in the polylinker of pME6014.

### Bacterial infiltration of rice leaves and RNA gel blot analysis

Bacterial strains were grown on a rotary shaker (220 rpm) over night at 28°C in LB containing, where appropriate, 10 μg/ml tetracycline. Bacteria were pelleted by centrifugation, washed twice in distilled water, resuspended in distilled water at an optical density at 600 nm (OD_600_) of 0.4 (approximately 10^8 ^cfu), and infiltrated into first leaves of 14-day-old rice plants (*Oryza sativa *cv. Loto; supplied by Terreni alla Maggia, Ascona, Switzerland) as described previously [23]. RNA was extracted from infiltrated leaves 16 h after infiltration and subjected to gel blot analysis using a ^32^P-labeled *Pir7b *cDNA probe (GenBank:Z34270[23]) according to standard procedures [29].

### HPLC analysis and mass spectrometry of syringolin A

To analyze conditioned media with regard to syringolin A content, *Pseudomonas *strains were grown in SRM_AF _medium [36,37] at 28°C for 60 h on a rotary shaker (220 rpm). Bacteria were pelleted by centrifugation and the supernatant was sterile filtered (0.22 μm pore size). Two-hundred-microliter aliquots were acidified with trifluoroacetic acid (TFA; 0.3% final concentration) and subjected to reverse-phase HPLC with a Reprosil 100-5 C_18 _250/4.6 column (Dr. Maisch GmbH, Ammerbuch-Entringen, Germany) on a Dionex UltiMate 3000 system (Dionex Corporation, Sunnyvale, CA). Elution was performed isocratically with 20% acetonitrile and 0.06% TFA in water at a flow rate of 1 ml/min.

High-resolution electrospray mass spectra were recorded on a Bruker maXis QTOF-MS instrument (Bruker Daltonics GmbH, Bremen, Germany). The samples were dissolved in MeOH and analyzed via continuous flow injection at 3 μl/min. The mass spectrometer was operated in positive ion mode with a capillary voltage of 4 kV, an endplate offset of -500 V, nebulizer pressure of 5.8 psig, and a drying gas flow rate of 4 l/min at 180°C. The instrument was calibrated with a Fluka electrospray calibration solution (Sigma-Aldrich, Buchs, Switzerland) that was 100 times diluted with acetonitrile. The resolution was optimized at 30'000 FWHM in the active focus mode. The accuracy was better than 2 ppm in a mass range between *m/z *118 and 2721. All solvents used were purchased in best LC-MS qualities.

### ^13^C-labeling and NMR Spectroscopy

*Pss *B301D-R was transformed with pOEAC and grown in LB containing 10 μg/ml tetracycline on a shaker at 28°C until an OD_600 _of approximately 0.5 was reached. Bacteria were collected by centrifugation, washed twice with SRM_AF _medium, and taken up in SRM_AF _medium at an OD_600 _of 0.3. Fifty-ml cultures were inoculated with 0.01 volume of the bacterial suspension and incubated at 28°C on a shaker (220 rpm). After 48 h, NaH^13^CO_3 _(98%; Sigma-Aldrich, Buchs, Switzerland) was added to a final concentration of 70 mM and incubation was continued for 20 h. Bacteria were pelleted and syringolin A was isolated from sterile-filtrated conditioned media as described [4].

^1^H broadband decoupled ^13^C NMR spectra were recorded at 25°C on a Bruker Avance III 600 MHz spectrometer equipped with a cryogenic 5 mm CPDCH probe head optimized for ^13^C detection. Two samples were prepared by dissolving 200 μg of labeled syringolin A in 130 μl DMSO-d6 and 5 mg of unlabeled syringolin A in 750 μl DMSO-d6, respectively. The labeled sample was transferred to a 3 mm Shigemi tube, the unlabeled sample was transferred to a regular 5 mm NMR tube. The spectral width in both spectra was 248.5 ppm, the transmitter was set to 100 ppm. The excitation pulse angle was set to 45°. The acquisition time was 2.1 s with a waiting time of 0.3 s between two scans. Both spectra were ^1^H broadband decoupled using the waltz16 composite-pulse decoupling scheme. The resulting fid consisted of 157890 total data points. For the unlabeled syringolin A sample 4000 scans were accumulated. For the labeled syringolin A sample 29605 scans were accumulated. Both spectra were zero filled to 131072 complex data points and processed using an exponential line broadening of 2 Hz. The samples contained no internal chemical shift reference and the spectra were referenced to the solvent peak (39.5 ppm). By comparison with chemical shifts listed in [1] the signals at 157.8 ppm and 132.8 ppm were assigned to the ureido CO group and the olefinic C at position 4 in the 3,4-dehydrolysine moiety, respectively.

## Authors' contributions

CR carried out the majority of experiments. The pPL3syl cosmid and the Δsyl deletion mutant were constructed by MT and MM, respectively. Mass spectrometry and NMR spectroscopy were performed and analyzed by LB and MOE, respectively. BS performed RNA gel blot analyses in the rice infiltration experiments. RD, CR, and BS designed experiments and RD wrote a draft manuscript. All authors provided critical inputs to the manuscript.

## References

[B1] Wäspi U, Blanc D, Winkler T, Ruedi P, Dudler R (1998). Syringolin, a novel peptide elicitor from *Pseudomonas syringae *pv. *syringae *that induces resistance to *Pyricularia oryzae *in rice. Mol Plant-Microbe Interact.

[B2] Wäspi U, Hassa P, Staempfli A, Molleyres L-P, Winkler T, Dudler R (1999). Identification and structure of a family of syringolin variants: Unusual cyclic peptides from *Pseudomonas syringae *pv. *syringae *that elicit defense responses in rice. Microbiol Res.

[B3] Michel K, Abderhalden O, Bruggmann R, Dudler R (2006). Transcriptional changes in powdery mildew infected wheat and Arabidopsis leaves undergoing syringolin-triggered hypersensitive cell death at infection sites. Plant Mol Biol.

[B4] Wäspi U, Schweizer P, Dudler R (2001). Syringolin reprograms wheat to undergo hypersensitive cell death in a compatible interaction with powdery mildew. Plant Cell.

[B5] Groll M, Schellenberg B, Bachmann AS, Archer CR, Huber R, Powell TK, Lindow S, Kaiser M, Dudler R (2008). A plant pathogen virulence factor inhibits the eukaryotic proteasome by a novel mechanism. Nature.

[B6] Clerc J, Groll M, Illich DJ, Bachmann AS, Huber R, Schellenberg B, Dudler R, Kaiser M (2009). Synthetic and structural studies on syringolin A and B reveal critical determinants of selectivity and potency of proteasome inhibition. Proc Natl Acad Sci USA.

[B7] von Döhren H, Dieckmann R, Pavela-Vrancic M (1999). The nonribosomal code. Chem Biol.

[B8] Marahiel MA, Stachelhaus T, Mootz HD (1997). Modular peptide synthetases involved in nonribosomal peptide synthesis. Chem Rev.

[B9] Finking R, Marahiel MA (2004). Biosynthesis of nonribosomal peptides. Annu Rev Microbiol.

[B10] Amrein H, Makart S, Granado J, Shakya R, Schneider-Pokorny J, Dudler R (2004). Functional analysis of genes involved in the synthesis of syringolin A by *Pseudomonas syringae *pv. *syringae *B301D-R. Mol Plant-Microbe Interact.

[B11] Fischbach MA, Walsh CT (2006). Assembly-line enzymology for polyketide and nonribosomal peptide antibiotics: Logic, machinery, and mechanisms. Chem Rev.

[B12] Hopwood DA (1997). Genetic contributions to understanding polyketide synthases. Chem Rev.

[B13] Harada K, Fujii K, Shimada T, Suzuki M, Sano H, Adachi K, Carmichael WW (1995). Two cyclic peptides, anabaenopeptins, a third group of bioactive compounds from the cyanobacterium *Anabaena flos-aquae *NRC-525-17. Tetrahedron Lett.

[B14] Murakami M, Shin HJ, Matsuda H, Ishida K, Yamaguchi K (1997). A cyclic peptide, anabaenopeptin B, from the cyanobacterium *Oscillatoria agardhii*. Phytochemistry.

[B15] Gesner-Apter S, Carmeli S (2008). Three novel metabolites from a bloom of the cyanobacterium Microcystis sp. Tetrahedron.

[B16] Okumura HS, Philmus B, Portmann C, Hemscheidt TK (2009). Homotyrosine-containing cyanopeptolins 880 and 960 and anabaenopeptins 908 and 915 from *Planktothrix agardhii *CYA 126/8. J Nat Prod.

[B17] Williams DE, Craig M, Holmes CFB, Andersen RJ (1996). Ferintoic acids A and B, new cyclic hexapeptides from the freshwater cyanobacterium *Microcystis aeruginosa*. J Nat Prod.

[B18] Matthew S, Ross C, Paul VJ, Luesch H (2008). Pompanopeptins A and B, new cyclic peptides from the marine cyanobacterium *Lyngbya confervoides*. Tetrahedron.

[B19] Schmidt EW, Harper MK, Faulkner DJ (1997). Mozamides A and B, cyclic peptides from a theonellid sponge from Mozambique. J Nat Prod.

[B20] Muller D, Krick A, Kehraus S, Mehner C, Hart M, Kupper FC, Saxena K, Prinz H, Schwalbe H, Janning P (2006). Brunsvicamides A-C: Sponge-related cyanobacterial peptides with *Mycobacterium tuberculosis *protein tyrosine phosphatase inhibitory activity. J Med Chem.

[B21] Staskawicz B, Dahlbeck D, Keen N, Napoli C (1987). Molecular characterization of cloned avirulence genes from race-0 and race-1 of *Pseudomonas syringae *pv. *glycinea*. J Bacteriol.

[B22] Wäspi U, Misteli B, Hasslacher M, Jandrositz A, Kohlwein SD, Schwab H, Dudler R (1998). The defense-related rice gene *Pir7b *encodes an "alpha/beta hydrolase fold" protein exhibiting esterase activity towards naphthol AS-esters. Eur J Biochem.

[B23] Reimmann C, Hofmann C, Mauch F, Dudler R (1995). Characterization of a rice gene induced by *Pseudomonas syringae *pv. *syringae*: Requirement for the bacterial *lemA *gene function. Physiol Mol Plant Pathol.

[B24] Senior E, Bull AT, Slater JH (1976). Enzyme evolution in a microbial community growing on herbicide Dalapon. Nature.

[B25] Smith JA, Métraux JP (1991). *Pseudomonas syringae *pathovar *syringae *induces systemic resistance to *Pyricularia oryzae *in rice. Physiol Mol Plant Pathol.

[B26] Kanehisa M, Araki M, Goto S, Hattori M, Hirakawa M, Itoh M, Katayama T, Kawashima S, Okuda S, Tokimatsu T (2008). KEGG for linking genomes to life and the environment. Nucleic Acids Res.

[B27] Golemi D, Maveyraud L, Vakulenko S, Samama JP, Mobashery S (2001). Critical involvement of a carbamylated lysine in catalytic function of class D beta-lactamases. Proc Natl Acad Sci USA.

[B28] Maveyraud L, Golemi D, Kotra LP, Tranier S, Vakulenko S, Mobashery S, Samama JP (2000). Insights into class D beta-lactamases are revealed by the crystal structure of the OXA10 enzyme from Pseudomonas aeruginosa. Structure.

[B29] Ausubel FM, Brent R, Kingston RE, Moore DD, Smith JA, Seidman JG, Struhl K (1987). Current protocols in molecular biology.

[B30] Finan TM, Kunkel B, Devos GF, Signer ER (1986). 2nd symbiotic megaplasmid in *Rhizobium meliloti *carrying exopolysaccharide and thiamin synthesis genes. J Bacteriol.

[B31] Christensen BB, Sternberg C, Andersen JB, Palmer RJ, Nielsen AT, Givskov M, Molin S (1999). Molecular tools for the study of biofilm physiology. Methods Enzymol.

[B32] Voisard C, Bull CT, Keel C, Laville J, Maurhofer M, Schnider U, Défago G, Haas D, F. OG, D. D, B B (1994). Biocontrol of root diseases by *Pseudomonas fluorescens *CHA0: current concepts and experimental approaches. Molecular ecology of rhizosphere microorganisms.

[B33] Simon R, Priefer U, Puhler A (1983). A broad host range mobilization system for *in vivo *geneic engineering: transposon mutagenesis in Gram-negative bacteria. Bio-Technology.

[B34] Blumer C, Heeb S, Pessi G, Haas D (1999). Global GacA-steered control of cyanide and exoprotease production in *Pseudomonas fluorescens *involves specific ribosome binding sites. Proc Natl Acad Sci USA.

[B35] Schnider-Keel U, Seematter A, Maurhofer M, Blumer C, Duffy B, Gigot-Bonnefoy C, Reimmann C, Notz R, Defago G, Haas D (2000). Autoinduction of 2,4-diacetylphloroglucinol biosynthesis in the biocontrol agent Pseudomonas fluorescens CHA0 and repression by the bacterial metabolites salicylate and pyoluteorin. J Bacteriol.

[B36] Gross DC (1985). Regulation of syringomycin synthesis in *Pseudomonas syringae *pv. *syringae *and defined conditions for its production. J Appl Bacteriol.

[B37] Mo Y-Y, Gross DC (1991). Plant signal molecules activate the *syrB *gene, which is required for syringomycin production by *Pseudomonas syringae *pv. *syringae*. J Bacteriol.

